# The Role of Plain CT in Assessing and Estimating Normal Values of Pericardial Fat Pad Thickness and Their Correlation With Patient’s Age, Gender, Body Weight, and Body Mass Index

**DOI:** 10.7759/cureus.66271

**Published:** 2024-08-06

**Authors:** Karthik Krishna Ramakrishnan, Michael Antony Vikram, Ajina Sam, Yuvaraj Muralidharan, Paarthipan Natarajan

**Affiliations:** 1 Radiodiagnosis, Saveetha Medical College and Hospital, Saveetha Institute of Medical and Technical Sciences (SIMATS), Saveetha University, Chennai, IND

**Keywords:** epicardial adipose tissue, south indian population, body weight influence, gender differences, gender correlation, age correlation, non-invasive imaging, cardiovascular risk, computed tomography, pericardial fat pad

## Abstract

Introduction

The pericardial fat pad, located anteriorly to the heart between the pericardium and myocardium, has garnered significant interest in cardiovascular research due to its potential role in the pathophysiology of various cardiac conditions. Despite its proximity to the myocardium, it is distinct from the epicardial fat depot found between the myocardium and the visceral layer of the pericardium. Studies have shown that excess pericardial fat is associated with an increased risk of heart failure and other cardiovascular diseases. Non-contrast computed tomography (CT) is a reliable, non-invasive method for assessing pericardial fat pad thickness, offering less radiation exposure compared to other imaging modalities. Establishing standardized measurements for pericardial fat pad thickness is essential, particularly for the South Indian population, which may exhibit unique genetic, dietary, and lifestyle influences on these measurements.

Materials and methods

A cross-sectional study was conducted on 300 participants from South India, stratified into three age groups: 18-35, 36-50, and 51-70 years, with body weights ranging from 45 kg to 120 kg. Participants were recruited from outpatient departments and community outreach programs, ensuring equal representation from each age group. Non-contrast CT imaging was performed using a Siemens Somatom goTop 128 Slice CT scanner to measure pericardial fat pad thickness and correlate it with age, gender, body weight, and body mass index. Exclusion criteria included diagnosed cardiac or pericardial diseases, prior chest surgery or trauma, pregnancy, and contraindications to CT scans. Ethical approval was obtained, and informed consent was collected from all participants. Data analysis was performed using SPSS software, employing descriptive statistics, ANOVA, t-tests, and Pearson's correlation.

Results

The study included 300 participants, with an equal gender distribution of 150 males and 150 females. Pericardial fat pad thickness increased with age, averaging 4.2 mm in the 18-35 age group, 5.1 mm in the 36-50 age group, and 6.4 mm in the 51-70 age group. Males exhibited a higher average thickness (5.6 mm) compared to females (5.0 mm). Body weight also showed a positive correlation with pericardial fat pad thickness, with mean values increasing from 4.5 mm in the 45-60 kg range to 6.7 mm in the 106-120 kg range. Statistical analysis confirmed significant differences in pericardial fat pad thickness across age groups, genders, and weight categories, emphasizing the importance of these factors in assessing cardiovascular risk.

Conclusion

This study provides a benchmark for pericardial fat pad thickness in the Kancheepuram Population of South India, highlighting its correlation with age, gender, body weight, and body mass index. The findings underscore the significance of non-invasive CT imaging in evaluating cardiovascular risk factors. Further research should focus on longitudinal studies and advanced imaging techniques to enhance the diagnostic accuracy and clinical relevance of pericardial fat pad measurements. The established reference values can aid clinicians in identifying individuals at higher risk for cardiovascular diseases, facilitating early intervention and management.

## Introduction

The pericardial fat pad is a deposit of adipose tissue located anteriorly to the heart consisting of two layers: the visceral, epicardial fat layer and the parietal, paracardial fat layer. Epicardial fat is the adipose tissue layer situated between the myocardium and visceral pericardium. The paracardial fat layer is the adipose tissue that accumulates outside the parietal pericardium [1]. This has garnered attention in cardiovascular research due to its possible relevance to the pathophysiology of many cardiac conditions.

Recent studies have highlighted the significant role of pericardial fat in various cardiovascular diseases. For instance, excess pericardial fat is associated with an increased risk of heart failure. Research by the National Heart, Lung, and Blood Institute (NHLBI) found that pericardial fat significantly increases the risk of heart failure, particularly heart failure with preserved ejection fraction, due to its direct impact on the stiffness of the heart muscle​ [2].

Additionally, the American College of Cardiology has reported that pericardial fat volume is linked to higher risks of coronary artery disease and myocardial dysfunction. It can secrete pro-inflammatory cytokines and adipokines, which contribute to inflammation and oxidative stress in the myocardium, thereby exacerbating cardiovascular conditions [3,4]. Hence, the quantification of the thickness of this particular structure may yield valuable insights regarding an individual's susceptibility to specific cardiovascular ailments. In addition, a study conducted by Thanassoulis et al. [5] revealed a positive correlation between augmented pericardial fat volume and the presence of calcium in coronary arteries, which serves as an indicator for the development of atherosclerosis. This observation suggests that pericardial fat might have a direct influence on the progression of atherosclerosis, regardless of the presence of other established risk factors.

The assessment of pericardial fat has predominantly been performed through the utilization of imaging modalities such as echocardiography, magnetic resonance imaging (MRI), and computed tomography (CT) scans. Among these options, CT provides a non-invasive, dependable, and replicable approach for measuring the thickness of the pericardial fat pad. In addition, the utilization of non-contrast CT scans has the supplementary benefit of less radiation exposure, rendering it a very suitable modality for conducting such assessments on a broad scale.

There is no substantial amount of literature available regarding the South Indian population, particularly that of the Kancheepuram District in India, characterized by its distinctive genetic composition, food practices, and lifestyle elements, which may demonstrate discernible variations in the thickness of pericardial fat. The population residing in South India is recognized for exhibiting an increased susceptibility to metabolic syndromes and cardiovascular diseases hence rendering this research particularly pertinent [6,7]. Therefore, the establishment of a standardized reference value within this specific demographic can assist healthcare professionals in categorizing individuals based on their risk levels and serve as a basis for conducting additional studies on the potential consequences of pericardial fat in this particular community.

This study aims to precisely assess the thickness of the pericardial fat pad in a sample of 300 individuals from the Kancheepuram region in South India, utilizing the Siemens Somatom goTop 128 CT scanner. By adopting a stratification approach, the measurements will be categorized into three distinct age groups (18-35, 36-50, and 51-70) to evaluate any discernible patterns or trends in pericardial fat pad thickness attributed to age. Additionally, the study seeks to conduct a comparative analysis between male and female participants to assess the potential influence of gender on the thickness of the pericardial fat pad. Another objective is to investigate the relationship between body weight (ranging from 45 kg to 120 kg) and pericardial fat pad thickness, shedding light on the influence of weight on this parameter. The data will be analyzed within the framework of established scholarly works to ascertain the therapeutic relevance of deviations in pericardial fat pad thickness, focusing on its predictive ability for cardiovascular risk. Ultimately, this research aims to establish a benchmark for pericardial fat pad thickness specific to the South Indian population, serving as a reference for future research and clinical assessments within this demographic.

## Materials and methods

**Figure 1:**
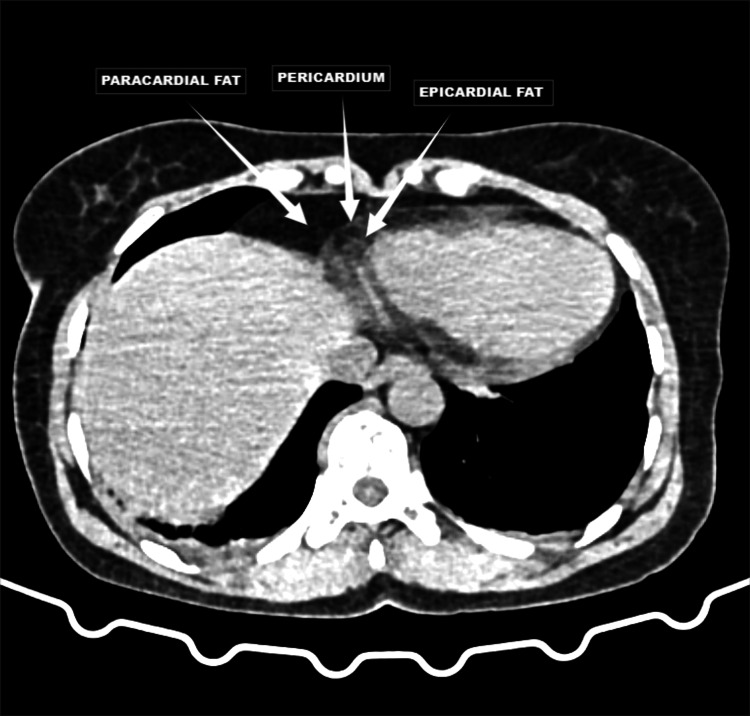
Representative axial non-contrast CT images demonstrating pericardial, paracardial and epicardial fat pads This figure highlights the pericardial fat composed of paracardial fat and epicardial fat separated by pericardium as labeled.

A cross-sectional study was conducted with a total of 300 participants at Saveetha Medical College and Hospital located in the suburbs of Chennai catering to the Kancheepuram population of South India, with 100 participants from each age group (18-35, 36-50, and 51-70) and patient weights ranging from 45 kg to 120 kg. These participants were subjected to non-contrast CT imaging to estimate the pericardial fat pad thickness and correlate it with age, gender, and body weight among the population of Kancheepuram population of South India. Participants were recruited from outpatient departments and through community outreach programs, utilizing a stratified random sampling technique to ensure equitable representation from each age group. Inclusion criteria included individuals aged 18-70 years, both genders and those willing to provide informed consent, while exclusion criteria involved individuals with diagnosed cardiac or pericardial diseases, prior chest surgery or trauma and participants with contraindications to CT scans like pregnancy. Figure 1 is a representative image that demonstrates the distinct locations of pericardial and epicardial fat, which is crucial for accurate measurement and assessment of pericardial fat pad thickness. This visualization aids in understanding the spatial relationship between these fat depots and the heart, enhancing the precision of our non-contrast CT imaging methodology.

**Figure 2:**
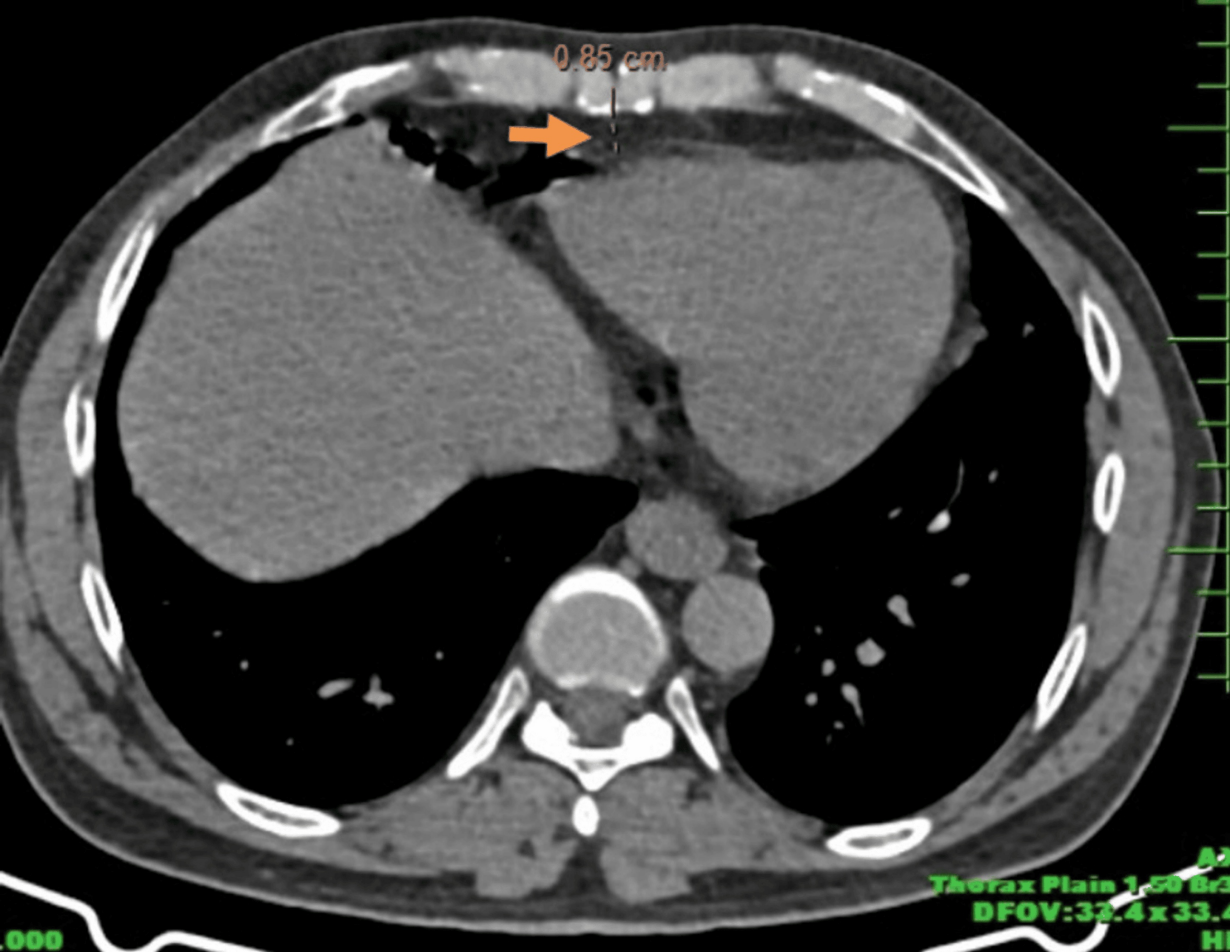
Axial section of CT thorax image showing the measurement of pericardial fat pad thickness (orange solid arrow)

The Siemens Somatom goTop 128-Slice CT scanner was used, with settings including a tube voltage of 120 kV, tube current of 250-300 mA, and a slice thickness of 2.5 mm to achieve optimal image quality. Patients were positioned supine with arms overhead to reduce artifacts, and AP and lateral scout images were taken to define the scan range from the carina to the cardiac apex. Axial slices were obtained at the level of the left major coronary artery, with windowing adjusted to optimize visualization of soft tissues as seen in Figure 2.

The study was conducted in strict adherence to the principles outlined in the Declaration of Helsinki and its subsequent revisions. The research protocol underwent thorough evaluation and obtained clearance from the Institutional Review Board (IRB) of Saveetha Medical College and Hospital, Saveetha Institute of Medical and Technical Sciences (SIMATS), Saveetha University (IRB reference number SMCH/SIMATS/2023-CT04). Participants provided written consent and confidentiality was maintained with anonymized data for analysis. Data analysis was performed using IBM SPSS Statistics for Windows, Version 23 (Released 2015; IBM Corp., Armonk, New York, United States), with descriptive statistics calculated for pericardial fat pad thickness. ANOVA was used to compare means across age groups, t-tests for gender differences, and Pearson's correlation for body weight relationships, with a p-value of <0.05 considered statistically significant. Quality control measures included training sessions for image interpretation consistency, standardized image acquisition protocols, and re-measurement of 10% of scans after a month to ensure reliability, measured by the intra-class correlation coefficient.

## Results

In a subset of our study involving 300 subjects using the Siemens Somatom goTop 128 CT scanner, we have provided insight into pericardial fat pad thickness in the South Indian sub-population from Kancheepuram. Participants were stratified based on age, gender, body weight, and BMI.

**Figure 3:**
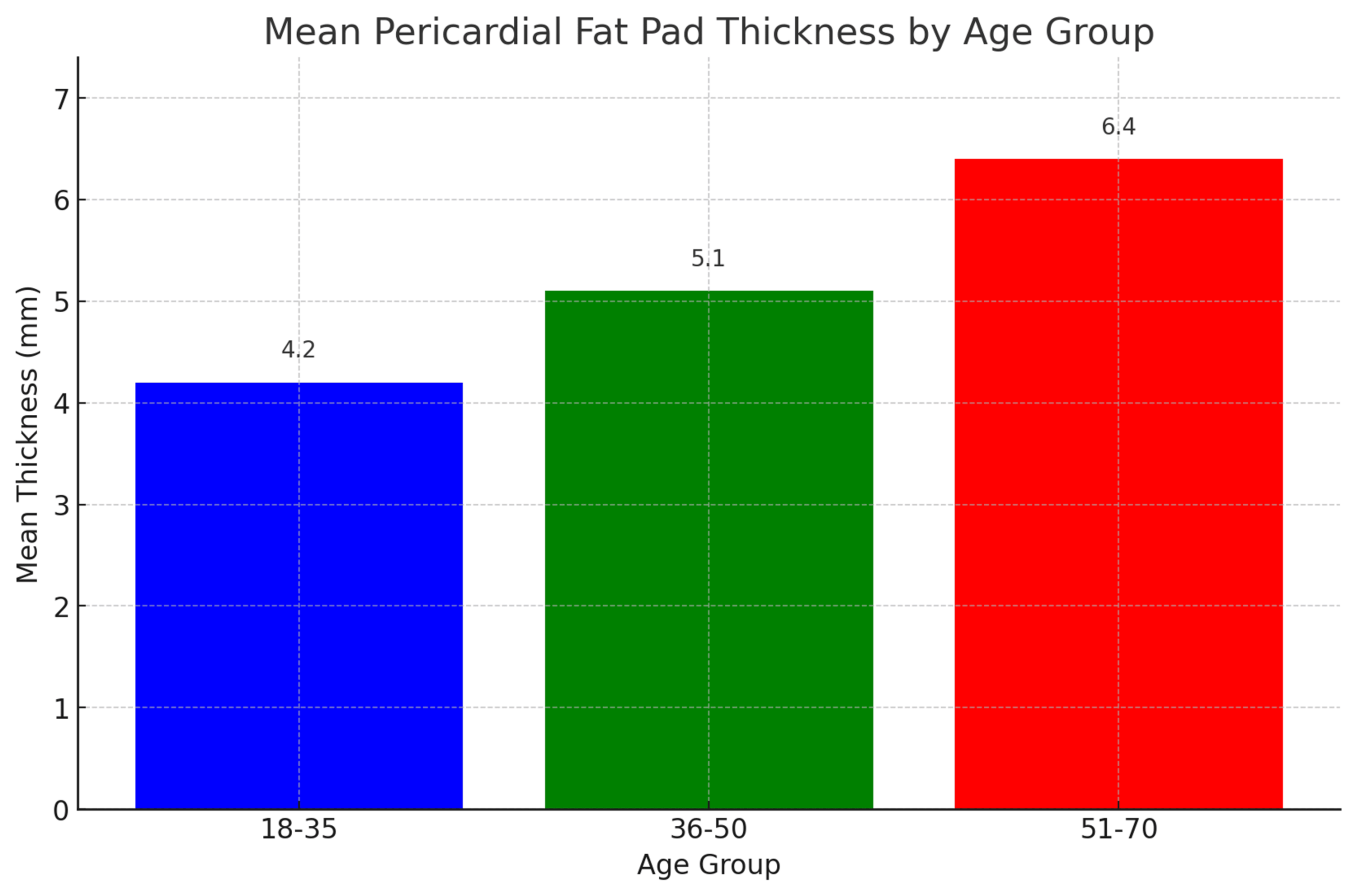
Mean pericardial fat pad thickness across different age groups

The study's findings on the distribution of pericardial fat pad thickness across different age groups reveal significant trends as seen in Table 1 and Figure 3, which summarizes the descriptive statistics for pericardial fat pad thickness among three age groups: 18-35, 36-50, and 51-70 years. The mean thickness of the pericardial fat pad increases with age, with the youngest group (18-35 years) having a mean thickness of 4.2 mm (SD = 0.8 mm), the middle age group (36-50 years) having a mean thickness of 5.1 mm (SD = 1.0 mm), and the oldest group (51-70 years) having the highest mean thickness of 6.4 mm (SD = 1.3 mm). The ANOVA test indicates that these differences are statistically significant (p < 0.001), suggesting that age is a significant factor influencing the thickness of the pericardial fat pad. This progressive increase in pericardial fat thickness with age could be attributed to several physiological changes associated with aging, such as alterations in fat distribution, increased adiposity, and metabolic changes.

**Table 1:** Descriptive statistics of pericardial fat pad thickness by age group ANOVA test revealed a significant difference in the mean thickness across age groups (p < 0.001).

Age group	No. of participants	Mean thickness (mm)	Standard deviation (mm)
18-35	100	4.2	0.8
36-50	100	5.1	1.0
51-70	100	6.4	1.3

**Figure 4:**
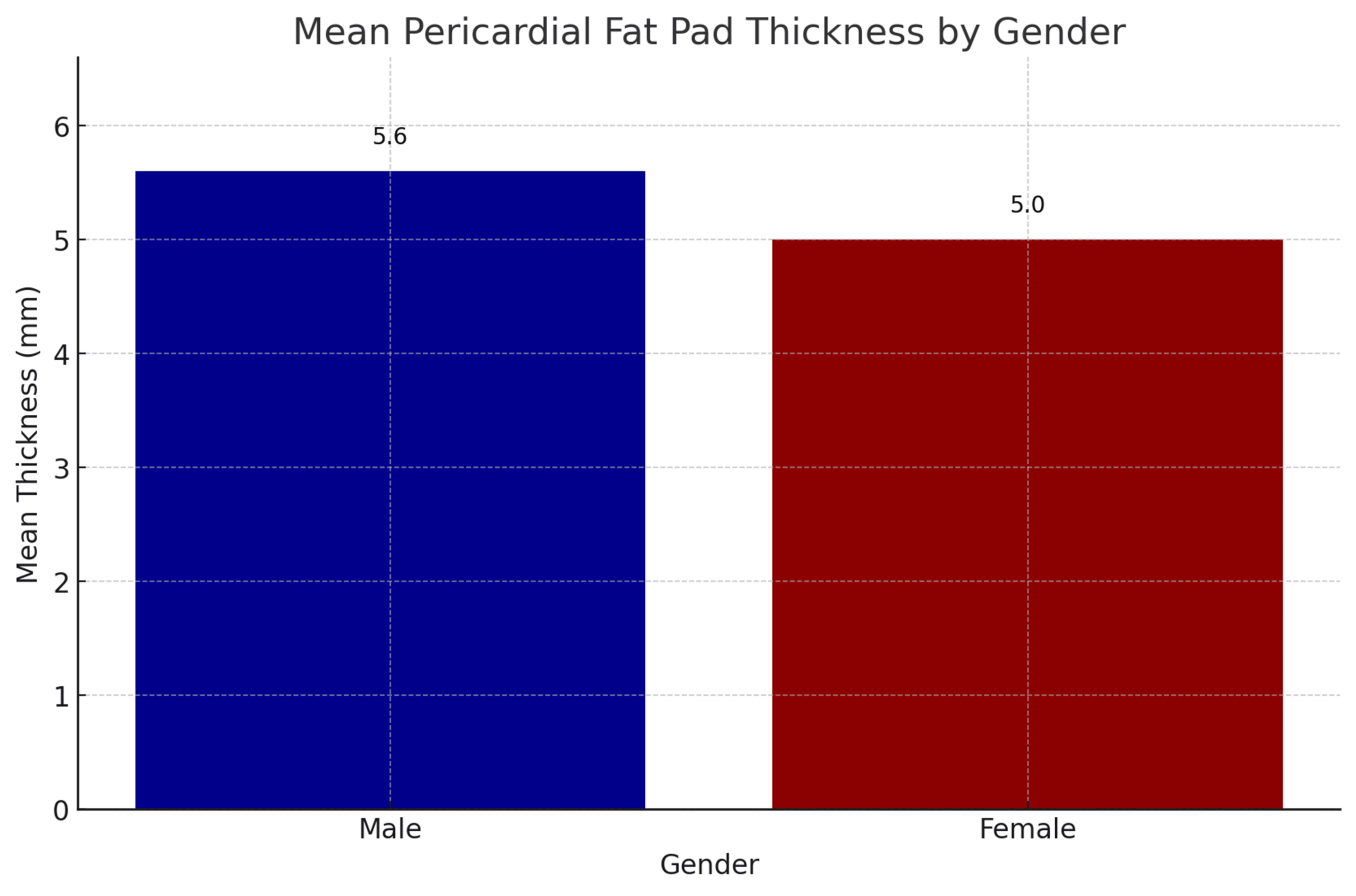
Mean pericardial fat pad thickness by gender

The study's findings on the gender distribution of pericardial fat pad thickness revealed notable differences between male and female participants as seen in Table 2 and Figure 4, which summarizes the descriptive statistics for pericardial fat pad thickness by gender, with 150 male and 150 female participants. The mean pericardial fat pad thickness for males is 5.6 mm (SD = 1.2 mm), while for females, it is 5.0 mm (SD = 1.1 mm). The t-test for independent samples indicates that the difference in mean pericardial fat pad thickness between males and females is statistically significant (p < 0.05). This result suggests that, on average, males have a higher pericardial fat pad thickness compared to females in this sample. The significant difference in pericardial fat pad thickness between genders could be attributed to various physiological and metabolic factors. Males tend to have higher overall body fat distribution in the visceral region, which may contribute to the observed differences in pericardial fat pad thickness.

**Table 2:** Descriptive statistics of pericardial fat pad thickness by gender Using the t-test for independent samples, there was a statistically significant difference in the pericardial fat pad thickness between males and females (p < 0.05).

Gender	No. of participants	Mean thickness (mm)	Standard deviation (mm)
Male	150	5.6	1.2
Female	150	5.0	1.1

**Figure 5:**
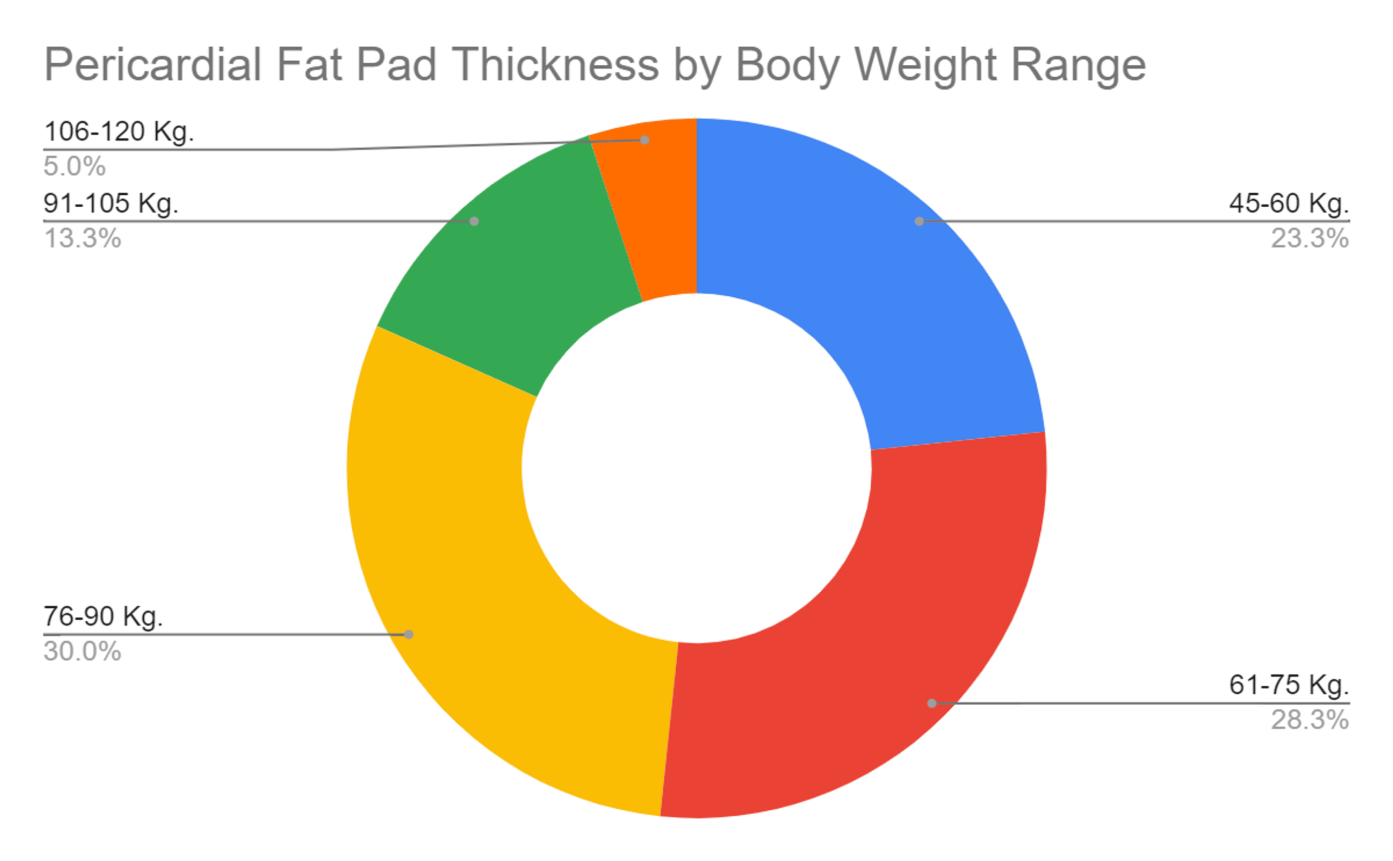
Mean pericardial fat pad thickness by body weight range

The study's analysis of pericardial fat pad thickness across different body weight ranges reveals significant variations and a positive correlation between body weight and fat pad thickness as seen in Table 3 and Figure 5 which presents the descriptive statistics for pericardial fat pad thickness categorized by body weight ranges: 45-60 kg, 61-75 kg, 76-90 kg, 91-105 kg, and 106-120 kg. For participants in the 45-60 kg range, the mean pericardial fat pad thickness is 4.5 mm (SD = 0.9 mm). In the 61-75 kg range, the mean thickness rises to 5.2 mm (SD = 1.0 mm), and further increases to 5.8 mm (SD = 1.2 mm) for those weighing 76-90 kg. Participants in the 91-105 kg range have a mean thickness of 6.1 mm (SD = 1.3 mm), and those in the highest weight range of 106-120 kg exhibit the greatest mean thickness of 6.7 mm (SD = 1.1 mm).

**Table 3:** Descriptive statistics of pericardial fat pad thickness by body weight range ANOVA test indicated a significant difference in the pericardial fat pad thickness across different weight ranges (p < 0.001). Further, a positive correlation was observed between body weight and pericardial fat pad thickness using Pearson's correlation test (r = 0.58, p < 0.001).

Body weight (kg)	No. of participants	Mean thickness (mm)	Standard deviation (mm)
45-60	70	4.5	0.9
61-75	85	5.2	1.0
76-90	90	5.8	1.2
91-105	40	6.1	1.3
106-120	15	6.7	1.1

The ANOVA test indicates that these differences in mean pericardial fat pad thickness across the various body weight ranges are statistically significant (p < 0.001). This finding suggests that body weight is a critical factor influencing the accumulation of pericardial fat. The increasing trend in pericardial fat pad thickness with higher body weight categories underscores the impact of adiposity on cardiac fat deposition. Additionally, Pearson's correlation test reveals a positive correlation between body weight and pericardial fat pad thickness (r = 0.58, p < 0.001). This significant correlation indicates that as body weight increases, there is a corresponding increase in pericardial fat pad thickness. This relationship highlights the potential risk that higher body weight poses for increased pericardial fat accumulation, which may contribute to adverse cardiovascular outcomes.

With respect to body mass index (BMI), the study's analysis showed a positive correlation between increasing BMI and pericardial fat pad thickness. The data presented in Table 4 highlights the relationship between BMI and pericardial fat pad thickness across different BMI categories. Participants were categorized into five BMI ranges as per the Asian criteria: <18.5 (underweight), 18.5-22.9 (normal), 23-24.9 (overweight), 25-29.9 (obese), and ≥30 (morbidly obese). The results show a clear trend of increasing mean pericardial fat pad thickness with higher BMI categories, with mean values of 3.7 mm, 4.4 mm, 5.3 mm, 6.2 mm, and 7.4 mm, respectively. The ANOVA test results in our study indicated a statistically significant difference in pericardial fat pad thickness across different BMI categories, with a p-value of less than 0.001. This low p-value signifies that the observed differences in mean pericardial fat pad thickness among the various BMI groups are unlikely to have occurred by chance, thereby confirming that BMI has a substantial impact on the accumulation of pericardial fat. Furthermore, Pearson's correlation test revealed a strong positive correlation (r = 0.58) between BMI and pericardial fat pad thickness. This correlation coefficient of 0.58 indicates a moderate to strong linear relationship, meaning that as BMI increases, pericardial fat pad thickness tends to increase correspondingly. These statistical findings underscore the critical role of BMI in influencing pericardial fat accumulation and highlight the importance of maintaining a healthy weight to mitigate cardiovascular risks associated with excessive pericardial fat.

**Table 4:** Descriptive statistics of pericardial fat pad thickness by body mass index

BMI category	Mean thickness (mm)	Standard deviation (mm)
Underweight (<18.5)	3.7	0.5
Normal (18.5-22.9)	4.4	0.5
Overweight (23-24.9)	5.3	0.6
Obese (25-29.9)	6.2	0.6
Morbidly obese (≥30)	7.4	0.7

## Discussion

Measurement of the pericardial fat pad thickness has evolved as a novel surrogate marker for understanding cardiovascular risk factors and risks associated with certain cardiac diseases. Our study from Kancheepuram was undertaken to explain the association between pericardial fat pad thickness with age, gender, body weight, and body mass index (BMI). Our study showed a progressively increasing trend in the pericardial fat pad thickness with increasing age mirroring the findings of a study in a Japanese cohort by Okura et al. [8]. There is no firm conviction of the exact mechanism behind this age-related increase. Declines in basal metabolic rate, hormone imbalances, and increased visceral fat deposition are all connected with aging and therefore may contribute to pericardial fat accumulation [4].

Concerning gender, our findings demonstrate that the pericardial fat pad was significantly thicker in males than in females. This agrees with a study done by Gorter et al. [9], where men had higher epicardial fat volume compared to women independent of BMI. Other studies, however, like the one conducted by Ahn et al. [10], revealed no significant differences between genders. It may be multifactorial-biased gender disparities regarding differences in hormonal profiles, genetics, and lifestyle factors from our study. Estrogen was suggested to exert a protective action on fat distribution, partly explaining the gender difference seen in our observation. Our study points to the apparent relationship between body weight and pericardial fat pad thickness, as viewed and supported in the literature. In one of these studies, Iacobellis et al. [11], indicated that epicardial fat relates to obesity parameters, including waist circumference, BMI, and visceral fat. This underlines the pathophysiological implications for the relationship between obesity and pericardial fat pad thickness. The proximity of the pericardial fat pad to the myocardium allows it to secrete pro-inflammatory cytokines directly into the coronary circulation, contributing to the development of coronary artery disease, diastolic dysfunction, and heart failure with preserved ejection fraction, with studies showing correlations between high pericardial fat volume and decreased diastolic function, increased left ventricular mass, and reduced left ventricular ejection fraction [3,4]. This increased pericardial fat is associated with systemic inflammation and insulin resistance, which are key components of metabolic syndrome. The correlation between BMI and pericardial fat pad thickness thus underscores the potential of this parameter as a marker for cardiovascular risk, particularly in the context of metabolic syndrome. 

The clinical relevance of measuring pericardial fat pad thickness can be explained by its probable relation to cardiovascular diseases. A study done by Rabkin [12], argued that the paracrine and endocrine functions can exert a function on myocardial function, causing atrial fibrillation. Moreover, Malavazos et al. [13], indicated that pericardial fat might have a more accurate prediction for coronary artery disease risk than even visceral abdominal fat.

Despite these insights, the study of pericardial fat has several limitations. The generalizability of findings is constrained by demographic and population-specific factors. Most studies, including those focused on specific regional populations like those in South India, may not be applicable to broader, more diverse populations. Additionally, the cross-sectional design of many studies limits the ability to establish causality. Longitudinal research is needed to better understand the progression and causal relationships between pericardial fat and cardiovascular diseases. Moreover, reliance on non-contrast CT imaging, while effective, may not fully capture the complexity of pericardial fat's impact. Advanced imaging techniques such as MRI could provide more detailed insights into the composition and physiological effects of pericardial fat.

Future research directions should include longitudinal studies to establish causative links between pericardial fat and cardiovascular outcomes. Tracking changes over time can provide more definitive evidence of the impacts of pericardial fat on heart health. Additionally, interventional studies that investigate the effects of targeted interventions, such as weight reduction programs or anti-inflammatory treatments, on pericardial fat and cardiovascular outcomes can offer actionable insights for clinical practice. Exploring these avenues will help develop more effective strategies for preventing and treating cardiovascular diseases related to pericardial fat.

## Conclusions

Our study, conducted in the culturally and genetically distinct region of Kancheepuram, South India, elucidates critical aspects of pericardial fat pad thickness and its associations with age, gender, and body weight. We have determined the precise range of pericardial fat pad thickness for the South Indian population, and we have observed a significant increase with age. Gender differences were noted, with males showing somewhat higher values than females. These observations offer subtle insights into physiological changes specific to a given community. The strong relationship between pericardial fat pad thickness and body weight highlights how important weight control is for cardiovascular health. These results have important clinical ramifications and provide a regional baseline of pericardial fat pad thickness that can be an invaluable diagnostic aid for physicians. Combining this measurement with conventional cardiovascular risk variables improves the evaluation of possible cardiac events or diseases. Moreover, the pericardial fat pad's metabolic activity and its connection to arrhythmias, coronary artery disease, and local inflammation highlight how crucial it is to comprehend the inflammatory and metabolic conditions of the pericardium.
